# Selection at a genomic region of major effect is responsible for evolution of complex life histories in anadromous steelhead

**DOI:** 10.1186/s12862-018-1255-5

**Published:** 2018-09-15

**Authors:** Steven J. Micheletti, Jon E. Hess, Joseph S. Zendt, Shawn R. Narum

**Affiliations:** 1 Columbia River Inter-Tribal Fish Commission, 3059F National Fish Hatchery Road, Hagerman, ID 83332 USA; 2 Columbia River Inter-Tribal Fish Commission, Portland, OR 97232 USA; 3 Yakama Nation Fisheries Program, Toppenish, WA 98948 USA

**Keywords:** *Oncorhynchus*, Adaptation, Genome evolution, Pooled sequencing, Sexual maturation, *greb1L*, Migration

## Abstract

**Background:**

Disparity in the timing of biological events occurs across a variety of systems, yet the understanding of genetic basis underlying diverse phenologies remains limited. Variation in maturation timing occurs in steelhead trout, which has been associated with *greb1L*, an oestrogen target gene. Previous techniques that identified this gene only accounted for about 0.5–2.0% of the genome and solely investigated coastal populations, leaving uncertainty on the genetic basis of this trait and its prevalence across a larger geographic scale.

**Results:**

We used a three-tiered approach to interrogate the genomic basis of complex phenology in anadromous steelhead. First, fine scale mapping with 5.3 million SNPs from resequencing data covering 68% of the genome confirmed a 309-kb region consisting of four genes on chromosome 28, including *greb1L*, to be the genomic region of major effect for maturation timing. Second, broad-scale characterization of candidate *greb1L* genotypes across 59 populations revealed unexpected patterns in maturation phenology for inland fish migrating long distances relative to those in coastal streams. Finally, genotypes from 890 PIT-tag tracked steelhead determined associations with early versus late arrival to spawning grounds that were previously unknown.

**Conclusions:**

This study clarifies the genetic bases for disparity in phenology observed in steelhead, determining an unanticipated trait association with premature versus mature arrival to spawning grounds and identifying multiple candidate genes potentially contributing to this variation from a single genomic region of major effect. This illustrates how dense genome mapping and detailed phenotypic characterization can clarify genotype to phenotype associations across geographic ranges of species.

**Electronic supplementary material:**

The online version of this article (10.1186/s12862-018-1255-5) contains supplementary material, which is available to authorized users.

## Background

Variation in the temporal occurrence of life history events, or phenology, occurs among a vast number of plant and animal systems [1–3]. The timing of processes such as migration, hibernation, flowering, and breeding can directly influence survival because essential resources vary over both time and space [4]. Therefore, maintaining interspecific phenological variation is often essential for a species’ persistence, as the timing of biological events may be beneficial or detrimental depending on cyclical variation of biotic and abiotic factors [5]. Thus, balancing selection can act to preserve variation in phenology [6, 7] which is often reflected by high genomic differentiation within multiple species [8, 9]. Consequently, discovery of phenology-related genomic variation is important both for understanding the genomic evolution of an organism and managing populations with phenological variation in the wild [10].

Variation in the timing of migration occurs across a variety of taxa but is particularly consistent and predictable in multiple anadromous salmonid species, which migrate from the ocean to freshwater tributaries to spawn [11]. Specifically, steelhead trout (*Oncorhynchus mykiss*) show distinct bimodal variation in the timing of entry into freshwater tributaries (freshwater entry maturation) [12, 13]. Steelhead that enter freshwater early are sexually premature (stream-maturing) and undergo maturation while in freshwater, whereas late migrating fish typically become sexually mature in the ocean prior to freshwater entry (ocean-maturing) [11, 14]. Despite these two distinct maturation-timing strategies, both stream and ocean-maturing fish spawn at similar times, and admixture occurs in coastal streams where both strategies are present [13, 15]. These alternate phenotypes do not affect population structure as fish with distinct maturation differences from the same geographic regions tend to be closely related across genome-wide markers [15–17]. In contrast to coastal populations, inland populations of steelhead are comprised exclusively of the stream-maturing type and enter freshwater sexually premature several months in advance of spawning as they migrate long distances to spawning tributaries [14, 16]. Even with similarity in maturation states at freshwater entry of inland populations of steelhead, uncertainty remains in whether variation in maturation exists near freshwater spawning grounds. Other salmonid species demonstrate temporal variation in maturation and arrival timing to inland spawning tributaries [18, 19] suggesting that variation in spawning site maturation may occur in steelhead.

The genetic basis for freshwater entry maturation in steelhead has recently been attributed to a single locus, *Growth regulation by estrogen in breast cancer-like* (*greb1L*), an oestrogen target-gene. Previous genomic reduction techniques (e.g., RAD-seq) identified multiple single-nucleotide polymorphisms (SNPs) within *greb1L* that successfully differentiated ocean and stream-maturing fish [13, 15]. However, these techniques were relatively coarse and effectively evaluated 0.5–2.0% of the steelhead genome, leaving uncertainty to the genetic basis of freshwater entry maturation. The recent availability of a high-quality *O. mykiss* genome assembly and full-genome resequencing techniques enables a more precise investigation of the genomic basis of maturation in steelhead. Further, access to passive integrated transponder (PIT) tagging data offers a proxy for measuring spawning site maturation that has not been explored in previous studies.

In this study we investigated three primary questions related to the evolution of complex phenology in anadromous steelhead. First, we evaluated whether dense genome scans could reveal additional candidate genes associated with stream versus ocean maturation phenotypes in steelhead from replicated streams. Second, we tested whether a previously identified candidate gene, *greb1L*, was consistently associated with freshwater entry maturation phenotypes in steelhead across a broad geographic range that included far-migrating inland populations in addition to previously studied coastal populations. Third, we examined spawning site arrival phenotypes from individual fish to test for genotype to phenotype associations of *greb1L* with spawning site maturation. Together, we synthesized results to explore models of selection that likely maintain genomic variation of complex maturation phenotypes across a broad distribution of steelhead.

## Methods

### Genomic resequencing for fine scale mapping of phenology traits

To acquire high-density coverage of the *O. mykiss* genome, we utilized a pooled-sequencing (Pool-seq) approach. Pool-seq involves combining individual DNA samples together and sequencing the homogenized DNA mixture together [20]. This method provides a representation of dense population allele frequencies across a reference genome [21]. However, dense representation of the genome comes at the loss of individual genotypes which are generally used to estimate metrics such as linkage disequilibrium and heterozygosity. We implemented Pool-seq in two independent spawning tributaries in the Columbia River Basin, the Klickitat and Kalama rivers, which both contain stream and ocean-maturing fish. We targeted fish for each library by peak run-times for the two phenologies: stream-maturing in July and ocean-maturing in March for total of 193 individuals collected between 2003 and 2005 (Kalama) and 2014–2017 (Klickitat). Using non-invasive samples of fin clips from fish trapped at weirs, we pooled samples accordingly: Kalama ocean-maturing samples (*n* = 50), Kalama stream-maturing samples (*n* = 46), Klickitat ocean-maturing samples (*n* = 47), Klickitat stream-maturing samples (*n* = 50) (Additional file 1: Table S1).

All four libraries were prepared using a modified NEBNext Ultra enzymatic fragmentation protocol (Additional file 1) [22, 23]. In summary, individual DNA was quantified with pico-green fluorescence on a Tecan M200 (Tecan, Männedorf, Switzerland) and normalized within two-standard deviations of the mean concentration to avoid over-representation of any given individual [23]. Pooled samples were fragmented using NEBNext Ultra dsDNA fragmentase and cleaned using a Qiagen MinElute. After ligation of Illumina adaptors, we targeted sequences with a mean size of 500 bp, performed PCR amplification, then cleaned PCR product using AMPure XP beads. All libraries were sequenced on an Illumina NextSeq 500 with a targeted 500–800 million paired-end reads per library.

### Pool-seq bioinformatics

Sequenced libraries were prepared with the *PoolParty* pipeline [23]. As part of this pipeline, raw 150 bp paired-end reads were filtered by trimming reads (to a minimum of 50 bp) with a base quality score less than 20 using the *trim-fastq.pl* script part of Popoolation [24]. Trimmed reads were then aligned to the *O. mykiss* reference assembly (Omyk_1.0; GCA_002163495) using bwa mem [25] with default parameters. PCR duplicates were removed using samblaster [26] and unpaired and unmapped reads were removed using the SAMtools *view* module [27]. Filtered BAM files were then combined using the SAMtools *mpileup* module, which extracts SNP and coverage information for each pool. To remove any false positive SNPs that often occur around insertion-deletions (indels) we used the *identify-genomic-indel-regions.pl* and *filter-sync-by-gtf.pl* scripts from Popoolation2 to remove any SNPs within 5 bp of indel regions [21]. Only variant positions with a minimum of 15 X depth of coverage and a maximum of 250 X depth of coverage were retained; this eliminated regions that may be paralogs (high coverage) or regions that were likely overrepresented by a small number of individuals (low coverage). Alignment and coverage statistics for all libraries were calculated using the *PPstats* module of *PoolParty*.

To determine selective sweeps or genomic regions with significant differentiation, we implemented sliding-window fixation index (F_ST_), a local score technique to test for statistical association, and a Cochran–Mantel–Haenszel (CMH) test [24, 28]. Sliding window F_ST_ between stream and ocean-maturing pools, was calculated using Popoolation2, using a sliding window of 5-kb with a step size of 50 bp [28]. Local score is an alternative to Fisher’s exact test (FET) for allele frequency difference that reduces false positives by incorporating linkage disequilibrium. Specifically, local score uses FET *p*-values to determine differentiated genomic regions while simultaneously considering linkage disequilibrium. Local score uses a score function related to -log(10) p which will vary based on window size. Opposed to combining *p*-values within a fixed window size, local score considers the proximity of statistically significant *p*-values to determine window size iteratively. Finally, the CMH test identifies consistent differences in allele frequencies across biological replicates and computes significance between groups of interest [29]. Thus, in our libraries, the CMH test identified SNPs with allele frequency changes that occurred between stream and ocean-maturing fish from both the Kalama and Klickitat. We considered genomic regions to be significant if they showed statistical support for differentiation between stream and ocean-maturing fish in both local score analyses (analogous to a Bonferroni corrected α = 0.05), and the CMH test (Bonferroni corrected α = 0.05). Significant regions were then investigated for variant annotations using *SnpEff* [22] which predicts non-synonymous SNPS (nsSNPs) from a general feature format (GFF). Variants identified as nsSNPs are anticipated to be under selection and more likely to be causal SNPs for a given trait [30].

Population structure analyses were performed using *PPanalyze* from the *PoolParty* pipeline. We used *PPanalyze* to remove SNPs with minor allele frequency (MAF) < 0.05 and create neighbor joining trees based on Nei’s genetic distance and 10,000 bootstraps, using both all genomic SNPs, and SNPs within a 309-kb region of chromosome 28.

### Broad scale characterization of candidate genotype frequencies

To determine association between maturation-timing and genomic regions of major effect across a large geographic scale, we isolated a single informative SNP within *greb1L* (*greb1L*-SNP) in an additional 59 collection localities across the Columbia River Basin [23] (*n* = 2915; Additional file 1: Table S3). In previous studies, *greb1L*-SNP explains a large proportion of trait variation in relation to maturation and consistently differentiates stream and ocean maturing fish [13]. Steelhead populations in North America are generally divided into coastal and inland genetic lineages [11, 31]. For example, the Columbia River Basin, which primarily encompasses the states of Oregon, Washington, and Idaho in the United States, consists of a coastal lineage west of the Cascade mountain range that is genetically distinct from an inland lineage east of the Cascades [32]. These two lineages also differ in respect to maturation-timing. The coastal lineage, which generally has shorter migration distances to spawning sites (50–380 km), consists of both stream and ocean-maturing fish. The inland lineage, which requires longer travel to spawning sites (370 –1500 km), only supports steam-maturing fish [18]. Due to the apparent lack of variation in maturation-timing in inland populations, previous studies have only investigated the genetic basis of steelhead migration and maturation in coastal populations [13, 15, 17]. However, we plotted genotype frequencies of *greb1L*-SNP across 59 collections including inland and coastal populations of steelhead using ArcGIS 10.5 to represent a broad geographic range.

### Individual phenotypes to refine genotype to phenotype associations

To determine an association between *greb1L*-SNP and spawning tributary arrival, we downloaded array ping dates from the Columbia Basin PIT Tag Information System (PTAGIS; ptasgis.org) for wild tagged fish between 2012 and 2016 in spawning tributaries across the Columbia River Basin. We retained data from sub-basins with known spawning tributaries which we also had sufficient individual DNA tissues (*N* > 50), leading to 6 distinct sub-basins, primarily in the inland lineage, with 890 fish in total (Additional file 1: Table S4). DNA from each individual was genotyped with a panel of markers using Genotyping-in-Thousands by sequencing (GT-seq) [33] to isolate and genotype a single *greb1L*-SNP (identified as 47080_54 in [13]). For each of the six sub-basins we determined significant associations between individual genotype (either premature [AA], heterozygote [AG], or mature [GG]) and spawning tributary arrival week using a one-way analysis of variance (ANOVA) paired with a Tukey’s range test [34]. We additionally determined the variance explained by the genotype in each location using ANOVA sum of squares.

## Results

### Genomic resequencing for fine scale mapping of phenology traits

**Fig. 1:**
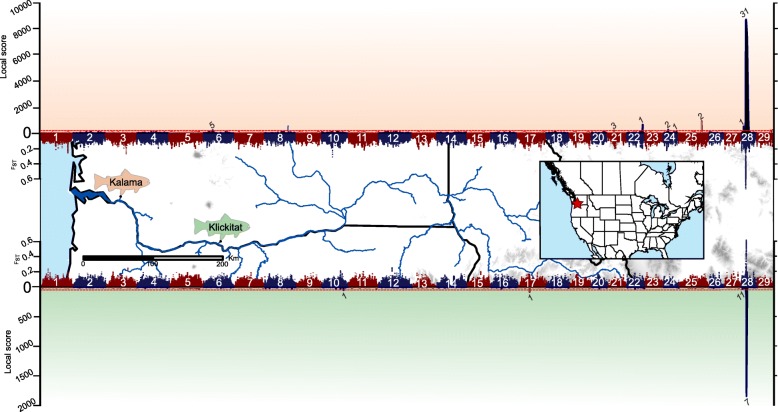
Genomic differentiation between ocean and stream maturing fish in the Kalama (top) and Klickitat (bottom) rivers in the Columbia River Basin [63–66]. Both Manhattan plots illustrate differentiation with sliding window F_ST_ (inward; 5 kb window) and local score (outward) across the 29 anchored *O. mykiss* assembly chromosomes. The red dotted line indicates a Bonferroni corrected threshold at α = 0.05. Numbers associated with significant peaks correspond to the number of annotates genes within the region (Additional file 1: Table S3)

**Fig. 2:**
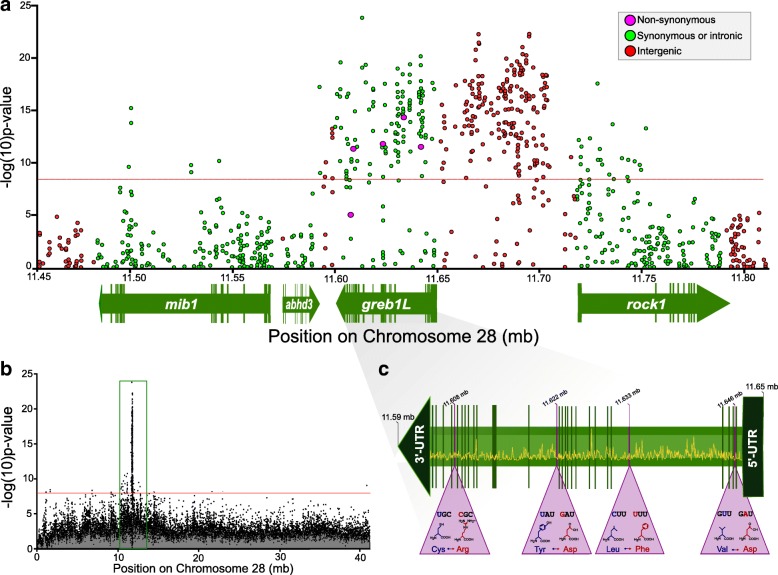
Genomic divergence based on a CMH test of chromosome 28 [63–66]. **a** Four annotated genes and intergenic region that are both significantly differentiated based on a CMH test with Bonferroni corrected threshold at α = 0.05. Green SNPs are those that are synonymous or fall outside of coding regions within genes, red SNPs correspond to intergenic or unannotated regions, and pink SNPs are non-synonymous mutations. Genes are displayed in their transcription orientation whereby arrows indicate 3’UTRs, bars represent other non-coding regions, open regions represent coding regions, and end-arrows represent 5’UTRs. **b** View of the entire chromosome 28 with CMH *p*-values and the differentiated region highlighted. **c** Magnification of the structure of *greb1L*, highlighting non-synonymous mutations. Pink triangles illustrate the amino acid substitution that occurs, whereby blue text represents the mutation in ocean-migrating fish and red text represents the mutation in stream-maturing fish. The yellow line represents SNP density at 5-kb windows across *greb1L*

Pooled-sequencing of four libraries consisting of ocean and stream-maturing fish from the Klickitat and Kalama rivers covered 68.2% of the *O. mykiss* assembly genome between 15 X – 250 X with an average depth of coverage of 33 X (Additional file 1: Figure S1). We identified a total of 21,957,491 SNPs between all four libraries that met coverage threshold criteria, among which 5,321,204 had a minor allele frequency (MAF) > 0.05. A comparison of ocean and stream-maturing fish between both populations using local score [35] and sliding-window F_ST_ [28] revealed that multiple genomic regions, consisting of 68 annotated genes in total, were significantly differentiated (Fig. 1; Additional file 1: Table S2). However, a single large genomic region on chromosome 28 was the only consistently significantly differentiated region between both comparisons. A Cochran–Mantel–Haenszel test (CMH test [29]) for consistent changes in allele frequencies between stream and ocean-maturing fish across both populations identified a highly differentiated 309-kb region consisting of 5,294 SNPs, 361 of which were statistically significant after a Bonferroni corrected threshold of α = 0.05 (Fig. 2a, b). When all genomic SNPs were considered, a neighbor-joining tree using Nei’s genetic distance [36] successfully separated libraries by the expected neutral geographic structure (Klickitat libraries vs. Kalama libraries; Additional file 1: Figure S2a). However, when only SNPs within the 309-kb region were considered, the neighbor-joining tree separated libraries by stream-maturing and ocean-maturing phenologies (Additional file 1: Figure S2b). Within the 309-kb significant region were four annotated genes: Mindbomb E3 Ubiquitin Protein Ligase 1 (*mib1mib1*), *Abhydrolase Domain Containing 3* (*abhd3*), *Growth regulation by estrogen in breast cancer-like 1*, (*greb1L*), and *Rho Associated Coiled-Coil Containing Protein Kinase 1*, (*rock1*), as well as two intergenic regions (Fig. 2a). While all genes contained significantly differentiated SNPs, *greb1L* and the immediate upstream intergenic region contributed the most significantly differentiated SNPs (36% and 51% of significant SNPs, respectively). Furthermore, a genomic variant annotation analysis using SnpEff [37] revealed that *greb1L* was the only gene to contain five non-conservative, non-synonymous SNP mutations (nsSNPs), four of which surpassed significance thresholds (Fig. 2c, Additional file 1: Table S3).

### Broad scale characterization of candidate genotype frequencies

**Fig. 3:**
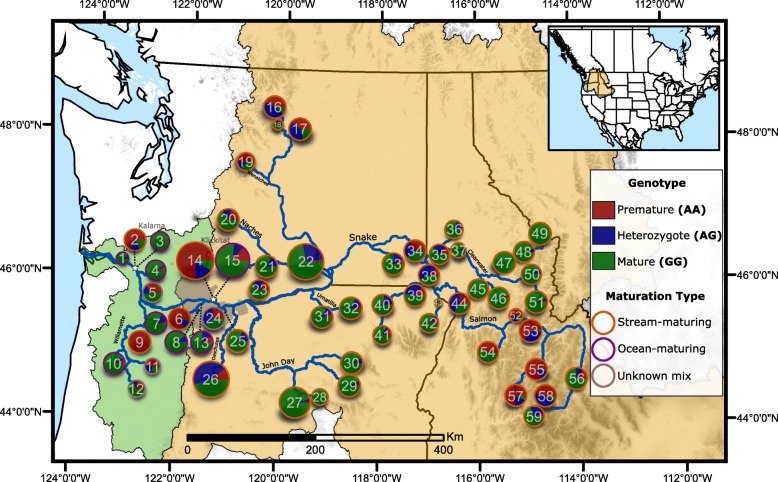
Map of the Columbia River Basin with 59 collection populations and their corresponding *greb1L*-SNP genotype composition [63–65]. For each population, the size of the circle corresponds to the number of sampled individuals, red corresponds to the premature genotype (AA), blue corresponds to heterozygous genotype (AG), and green corresponds to a mature genotype (GG). Population IDs correspond with Additional File 1: Table S4. Both the Klickitat and Kalama samples are divided by stream and ocean-maturing fish sampling. Within the Columbia River Basin, green map shading corresponds to the coastal lineage, whereas yellow map shading corresponds to the interior lineage. Grey shading between green and yellow indicate an admixture region between coastal and interior lineages

To determine the association between the differentiated chromosome 28 region with phenology on a broad geographic scale, we examined genotype frequencies of a diagnostic *greb1L* marker (*greb1L*-SNP; Chr.28, position 11613335) across both coastal and inland populations. This *greb1L*-SNP has been demonstrated to be strongly associated with freshwater entry maturation in previous studies of coastal steelhead populations [13, 15, 17]. Specifically, a “mature” *greb1L*-SNP genotype (GG) was strongly associated with ocean-maturation and a “premature” genotype (AA) was strongly associated with stream-maturation. Genotypes for the *greb1L*-SNP were isolated from a recent study using RAD-seq in 59 populations of steelhead across the Columbia River Basin [21] (Additional file 1: Table S4). Of the 59 populations, 15 were located in coastal streams, which support both ocean and stream-maturing fish, and 44 were from inland populations that consist solely of stream-maturing fish. In coastal populations, “premature” (AA) and “mature” genotypes (GG) were associated with stream-maturation and ocean-maturation, respectively (χ2 = 195, *p* < 0.001; Fig. 3). It was not possible to test for genotype-phenotype association of freshwater entry maturation in inland populations since they were all stream-maturing (*p* = 1); however, genotype frequencies were variable and the majority genotype in this region was the “mature” type (GG; x̅ = 65%). Prevalence of the “mature” genotype for inland populations was contrary to expectations for stream-maturing steelhead and suggested that further refinement of phenology phenotypes was necessary beyond a simplistic categorization of these populations into ocean and stream-maturing types.

### Individual phenotypes to refine genotype-by-phenotype associations

**Fig. 4:**
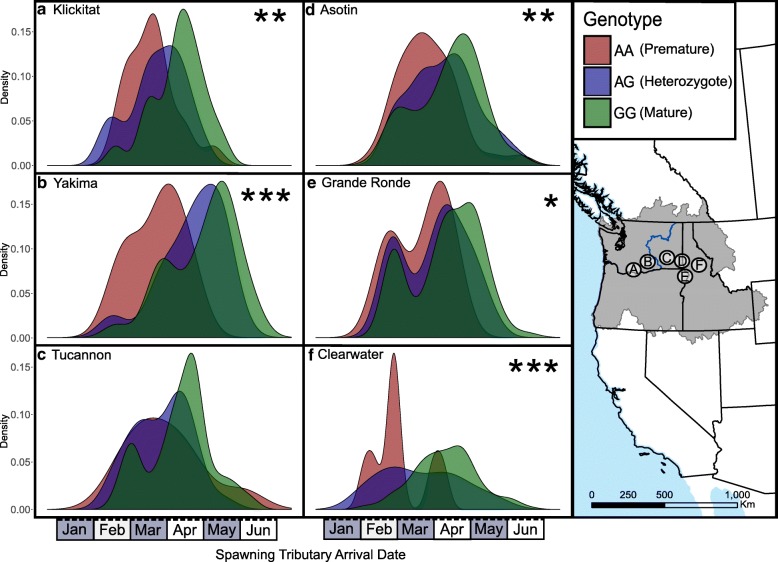
Density plots illustrating spawning tributary arrival date of fish with different maturation genotypes across spawning tributaries in 6 sub-basins: **a**) Klickitat, **b**) Yakima, **c**) Tucannon, **d**) Asotin, **e**) Grande Ronde, and, **f**) Clearwater. Asterisks in each panel correspond to the significance level between premature and mature genotypes (Tukey’s test): * = *p* < 0.05; ** = *p* < 0.01, *** = *p* < 0.001

We genotyped *greb1L*-SNP from 890 individual steelhead that were tracked throughout their migration to spawning tributaries in six sub-basins with PIT-tag arrays (ptagis.org) to test whether the chromosome 28 candidate region was more strongly associated with stream-maturing than ocean-maturing phenotype (Additional file 1: Table S5). Although the state of maturation of an individual fish is a challenging trait to measure, arrival timing to spawning sites at the end of freshwater migration can serve as a sufficient proxy for maturation [38, 39]. We used a one-way ANOVA paired with a Tukey post-hoc test to determine significant associations between *greb1L*-SNP genotypes and spawning tributary arrival date. All six sub-basins showed a trend between spawning tributary arrival date and genotype class, with five out of the six having statistically different arrival week means between “premature” and “mature” genotypes (Fig. 4, Additional file 1: Figure S3 and Table S5). On average, fish with “premature” genotypes arrived to spawning tributaries 3.04 ± 1.4 weeks prior to those with “mature” genotypes, fish with heterozygote genotypes arrived 1.64 ± 1.2 weeks later than those with “premature” genotypes and 1.51 ± 0.74 weeks earlier than those with “mature” genotypes. Across all six sub-basins genotypes from this single marker (*greb1L*-SNP) explained 10% ± 4% of spawning tributary arrival date variation (Additional file 1: Table S5).

## Discussion

Our study confirms a genomic region of major effect underlying phenological variation in anadromous steelhead through dense genome resequencing. Previous studies have mapped SNPs generated from restriction-site associated DNA sequencing (RAD-seq) to *greb1L*, yet have not explicitly explored additional genes of smaller effect upstream and downstream of *greb1L* [13, 15, 17]. Using dense mapping data, we illustrated that *greb1L* is part of a larger genomic region under selection consisting genes and divergent inter-genic regions. This discovery was made possible by both the advancement in quality of the *O. mykiss* genome assembly, and through resequencing techniques (Pooled-sequencing) that provide adequate read coverage across the majority of the reference genome [20]. While previous genome scans with RAD-seq yielded significant association of markers from the *greb1L* region, fine scale mapping provided a broader understanding of the genomic basis of this phenological trait in steelhead due to higher marker density covering a large portion of the genome [40].

The additional genes of smaller effect characterized in this study indicate that the genomic basis for maturation phenology in anadromous steelhead may encompass a larger genomic region on chromosome 28 than previously understood. *greb1L* has consistently shown the most compelling associations to maturation phenotypes [13, 15, 17]. Our results additionally highlight extreme differentiation in the upstream intergenic region of *greb1L* which likely contains many regulatory components such as transcription factors, promoters, and enhancers [41]. *greb1L* has obvious connections to sexual maturation since it mediates the interaction of oestrogen with other target proteins [13, 42]. Migrating fish, either mature, or nearing maturity, have elevated levels of oestrogen in their bloodstream which relates to multiple sexual characteristics such as egg formation and testicular development [43]. Additionally, we showed that *greb1L* contains multiple non-conservative and non-synonymous mutations. These changes in protein structure are compelling candidates that are possibility under selection, which additionally provide evidence that *greb1L* is a key gene under selection for maturation phenology [30, 44]. *rock1*, the gene directly upstream of *greb1L*, also has obvious ties to maturation as it has been connected to embryo development in zebrafish [45] and testicular development in humans [46]. Furthermore, it is a key regulator of actin-myosin contraction [47] which may be connected to the long migration distances anadromous steelhead need to swim to reach spawning grounds [21]. *abhd3*, the gene immediately downstream from *greb1L*, was the least differentiated, yet is a physiological regulator of medium-chain phospholipids [48]. The necessity for efficient fat disposition is essential in fish during long-distance migration [19, 49]. *mib1* has less-apparent connections to migration and maturity as it primarily relates to cell apoptosis [50]; however, *mib1* does influence ventricle formation and can be connected to cardiac function and swimming performance [51, 52]. Finally, a large intergenic region is highly differentiated between *greb1L* and *rock1* which may simply be a gene that currently lacks annotation, or possibly a region that consists of enhancers or promoters for the nearby genes or non-coding RNAs. If the latter, these intergenic SNPs may play a large regulatory role in expression level [53]. Overall, this highly divergent region on chromosome 28 contains compelling candidate genes which justifies further validation and marker development to investigate maturation phenotypes of steelhead in more detail. For example, informative SNPs from these candidate genes can be included into high-throughput amplicon sequencing panels to screen large numbers of individuals [33].

Our results indicated that the candidate region on chromosome 28 is most consistently associated with arrival timing on spawning grounds rather than arrival timing at freshwater entry across populations included in this study. Traditionally, arrival timing at freshwater entry has been a phenotypic proxy for maturation timing that has been used to characterize fish entering freshwater as either sexually mature or premature [11], which partly owes to the relative ease of recording this proxy trait for returning steelhead at many lower river collection sites including dams, weirs, and hatcheries [54, 55]. Coastal tributaries support both ocean and stream-maturing fish, whereas steelhead returning to inland tributaries are all stream-maturing fish [18]. Previous studies have solely investigated freshwater entry maturation of steelhead in coastal and lowland rivers [13, 15, 17], and their findings beget expectations that all inland fish may be fixed for “premature” *greb1L* genotypes. To the contrary, using spawning tributary arrival time, we show that variation in maturation phenology does occur in inland steelhead, which are all stream-maturing fish. Specifically, about 10% of variance of time to arrival is explained by *greb1L-*SNP. While the strength of this pattern is not overwhelming, it is still compelling given that PIT-tag arrival times were used as a proxy and may be prone to some error, and only a single informative SNP was used. In addition, it currently serves as the only explanation for variation in *greb1L* genotypes in stream-maturing fish. Given this, fish with “premature” *greb1L*-SNP genotypes generally arrive to spawning tributaries early, fish with “mature” genotypes arrive later, and heterozygous fish arrive in an intermediate timeframe. This association suggests that inland fish tend to hold in larger freshwater tributaries for several months as premature fish, and then migrate to spawning grounds in headwater tributaries over a continuum of maturation states before all fish become sexually mature and spawn together. This admixture of fish with varying phenotypes shows no Wahlund effect heterozygote deficit [21], providing addition evidence that population structure is not directly influenced by this phenology [15]. Inland steelhead with “premature” genotypes ascend to spawning grounds early (premature arrival) and continue to mature there, whereas fish with “mature” genotypes become sexually mature in freshwater downstream of spawning grounds, then move upstream to spawning grounds once they are mature (mature arrival). However, in coastal populations, mature arrival and premature arrival are likely analogous to ocean-maturing and stream-maturing phenotypes (respectively) that are commonly observed as steelhead enter freshwater systems near the ocean. Coastal and inland steelhead populations contain *greb1L* “mature” and “premature” genotypes; however, the inland *greb1L* “mature” and “premature” genotypes both exhibit an early and narrow range of freshwater entry timings, but later become diverged in their spawning tributary entry timing. In contrast, the *greb1L* “mature” and “premature” genotypes of the coastal steelhead populations instead show large disparity in freshwater entry timing from the ocean. This difference in freshwater entry timings of the *greb1L* mature genotypes of the coastal versus inland lineages may be due to the long migration distance that inland fish must swim to reach their (300–1500 km) spawning sites [21]. Regardless of their *greb1L* genotypes, all fish from inland populations must migrate early to approach spawning grounds before environmental conditions become unfavorable [56, 57] including passage through unfavorable migratory corridors [21]. Then, when near inland spawning sites, balancing selection may preserve the variation in *greb1L* genotypes which manifests as variation in spawning tributary arrival (i.e.*,* premature or mature spawning tributary arrival). In some years, due to resources such as spawning habitat availability, and environmental conditions, it may be beneficial to arrive to a spawning tributary early (*greb1L* premature genotype), whereas in others it may be more beneficial to arrive late (*greb1L* mature genotype) [14, 58]. In addition, the benefits of tributary arrival may vary based on geographic localities. Thus, balancing selection likely maintains the genomic variation in chromosome 28, a phenomenon that may be confirmed with further studies that investigate variation in steelhead phenology in specific locations and throughout the species range.

As commonly illustrated, maintaining adaptive genetic variation is essential for protecting species at risk to extinction [59, 60]. We provide evidence that genomic variation linked to sexual maturation is present in inland steelhead populations, which were previously assumed to be fixed for *greb1L* premature genotypes similar to fish exhibiting stream-maturing phenotypes in coastal populations. This discovery illustrates that understanding complex phenology patterns is a difficult process due to challenges of monitoring migrating fish through sexual development stages across large watersheds. However, this challenge can be overcome by monitoring efforts that apply tags and collect non-lethal tissue from migrating fish as they enter freshwater [13]. A profound understanding of complex life history traits can enable managers to maintain the necessary levels of neutral and adaptive genetic variation in wild populations to mitigate impacts of climate change on complex phenology traits [15, 61, 62].

## Conclusions

Anadromous salmonids, a recreational and culturally significant family of fishes, have complex life histories related to migration and maturation. Previous investigations into the genetic basis of maturation-timing suggested that a single gene, *greb1L*, was related to sexually premature and mature entry of steelhead into freshwater systems. Using genome resequencing for fine scale mapping of maturation traits during migration, we identified a 309-kb genomic region of major effect for freshwater entry maturation that included four candidate genes, *greb1L*, *rock1*, *mib1*, and *abhd3*. This region also includes a highly significant intergenic region between *greb1L* and *rock1* which may play a regulatory role in expression of these genes. Additionally, broad-scale SNP genotypes from *greb1L* in populations and individuals refined genotype to phenotype associations, revealing that candidate genotypes were more consistently associated with timing of arrival on spawning grounds rather than freshwater entry maturation. These results suggest that there are fitness benefits to arriving to spawning tributaries prematurely or maturely, and variation in this trait is likely to be maintained by balancing selection. Together this study illustrates the importance of high precision genomic scans and detailed phenotypes to identify targets of selection.

## Additional file

Additional file 1: Supporting figures. Supporting tables. Pooled-sequencing protocol. (DOCX 820 kb)

## Abbreviations

*abhd3*: Abhydrolase Domain Containing 3
ANOVA: Analysis of Variance
CMH: Cochran–Mantel–Haenszel
FET: Fisher’s Exact Test
FST: Fixation Index
*greb1L*: Growth Regulation By Estrogen In Breast Cancer 1 Like
GT-seq: Genotyping-In-Thousands by sequencing
MAF: Minor Allele Frequency
*mib1*: Mindbomb E3 Ubiquitin Protein Ligase 1
PCR: Polymerase chain reaction
PIT: Passive Integrated Transponder
RAD-seq: Restriction Site Associated DNA Sequencing
*rock1*: Rho Associated Coiled-Coil Containing Protein Kinase 1
SNP: Single-Nucleotide Polymorphism
