# Supplementary material for: Selection at a genomic region of major effect is responsible for evolution of complex life histories in anadromous steelhead
Source: BMC Evol Biol. 2018 Sep 15;18:140. doi: 10.1186/s12862-018-1255-5 (PMC6139179; doi:10.1186/s12862-018-1255-5)
Supplement: Supplementary file 1 — Supporting figures. Supporting tables. Pooled-sequencing protocol. (DOCX 820 kb) [file 12862_2018_1255_MOESM1_ESM.docx]

**Additional File 1: Supporting Figures**


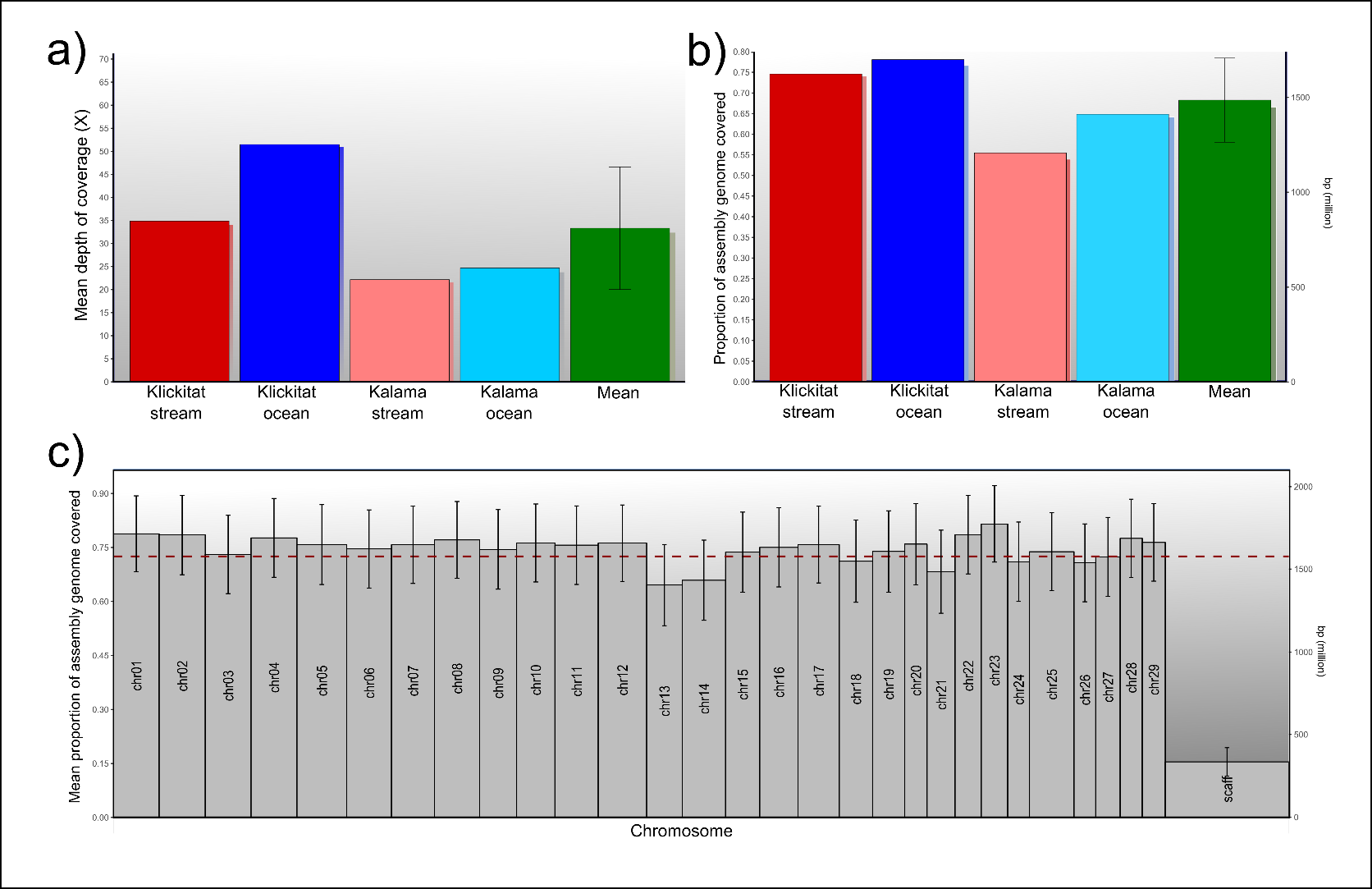


**Figure S1.** Coverage statistics for the four pooled-sequencing libraries. a) The mean depth of coverage of all aligned reads after quality filtering. b) The proportion of the full assembly genome covered by reads after quality filtering. c) The mean proportion of each chromosome and unanchored scaffolds combined covered by reads from all four libraries after quality filtering; the red dotted line indicates the mean of all chromosomes and scaffolds combined.


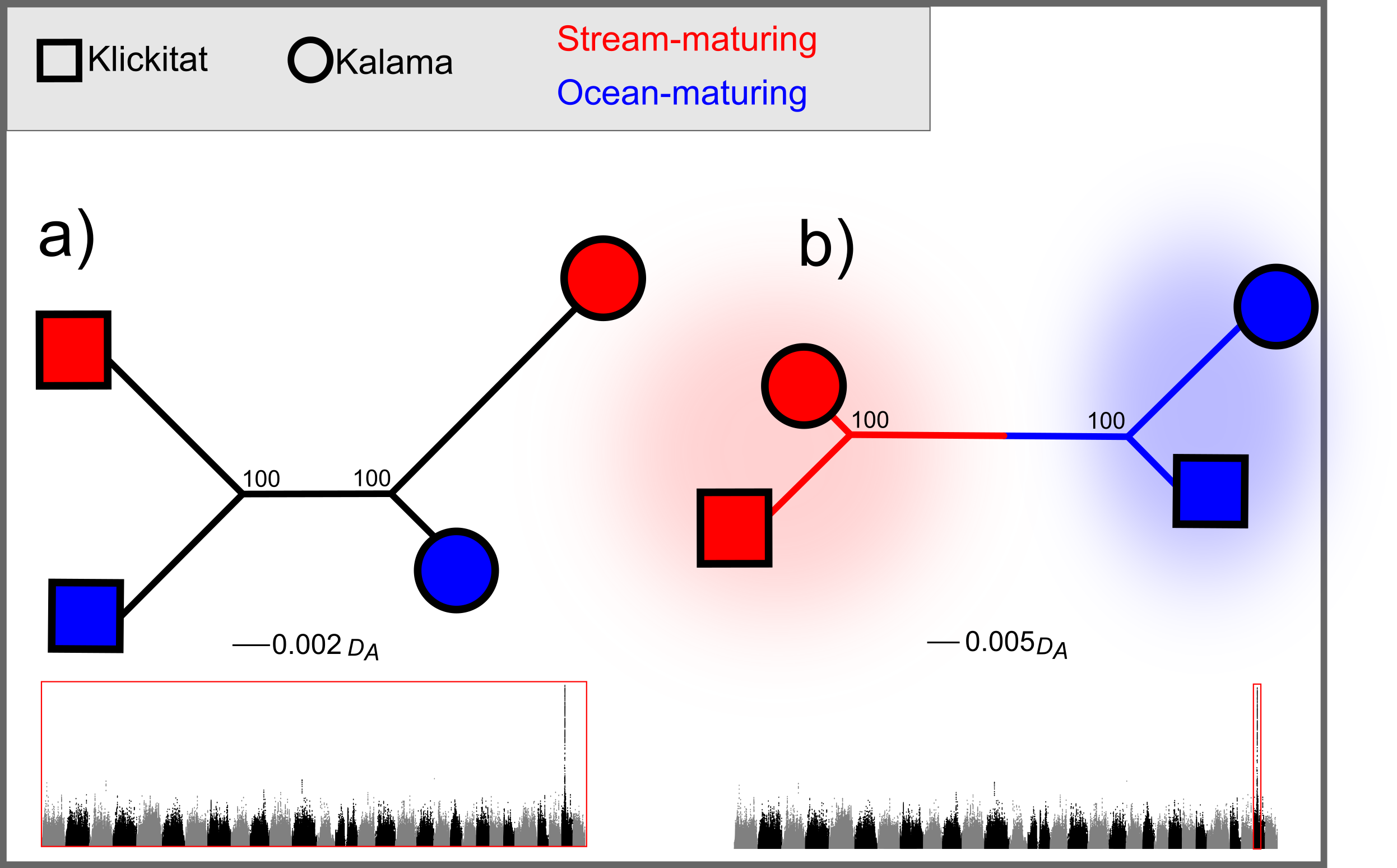


**Figure S2.** Neighbor-joining trees for a) all 5,321,204 genome-wide SNPs and b) the 5,294 SNPs within the differentiated region on chromosome 28. Squares represent Klickitat libraries, circles represent Kalama libraries, red represents stream-maturing libraries, and blue represents ocean-maturing libraries.


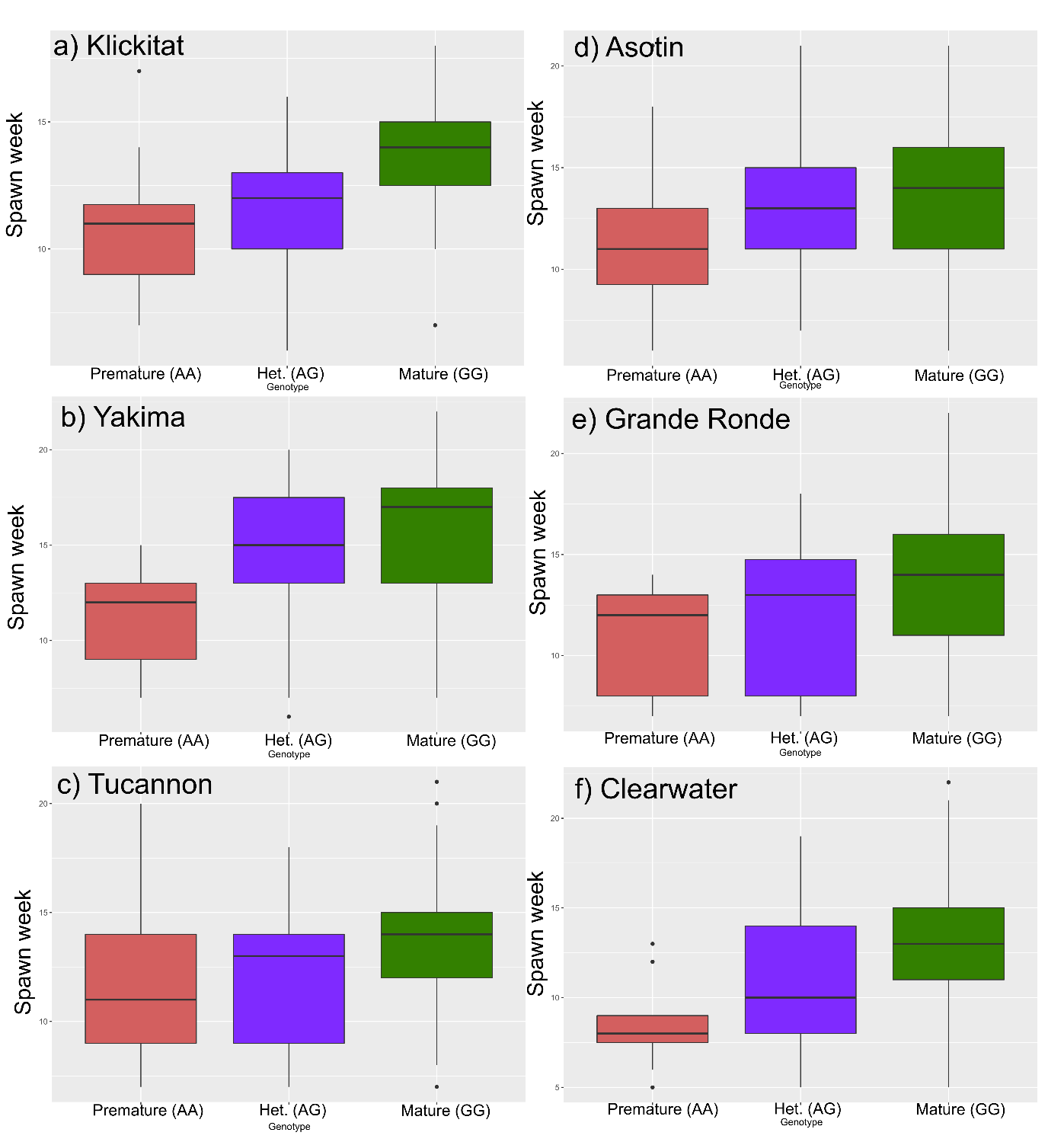


**Figure S3.** Boxplots indicating the association between greb1L-SNP genotypes and spawn week for 6 sub-basins.

**Additional File 1: Supporting Tables**

| Library | Tributary | Maturation | n | Reads (million) | Mean depth (X) | Prop. Cov. | Long | Lat |
| --- | --- | --- | --- | --- | --- | --- | --- | --- |
| 1 | Klickitat | Stream | 50 | 512.38 | 34.87 | 74.60% | -121.26 | 45.72 |
| 2 | Klickitat | Ocean | 50 | 753.49 | 51.49 | 78.06% | -121.26 | 45.72 |
| 3 | Kalama | Stream | 46 | 271.27 | 22.15 | 55.47% | -122.87 | 46.03 |
| 4 | Kalama | Ocean | 47 | 323.63 | 24.72 | 64.85% | -122.87 | 46.03 |

**Table S1.** Four pooled-sequencing libraries, the number of individuals (n) in each library and sequencing statistics. Reads corresponds to the number of reads after quality trimming. Mean depth and Prop. Cov. correspond to the mean depth of coverage and proportion of the assembly genome covered after quality filtering.

| **CHR** | **5' pos** | **3' pos** | **Length (bp)** | **Ori.** | **Gene ID** | **Gene** |
| --- | --- | --- | --- | --- | --- | --- |
| 6 | 24464475 | 24464899 | 424 | - | CIGENEomyV6.20479 | BMP1_HUMAN |
| 6 | 24474077 | 24484217 | 10140 | - | CIGENEomyV6.20483 | KANL3_DANRE |
| 6 | 24494583 | 24497312 | 2729 | - | CIGENEomyV6.20484 | MSPA_SAGOE |
| 6 | 24494656 | 24518222 | 23566 | + | CIGENEomyV6.20485 | TC1A_CAEEL |
| 6 | 24519433 | 24529046 | 9613 | + | CIGENEomyV6.20486 | ANTR1_MOUSE |
| 10 | 56720416 | 56720604 | 188 | + | CIGENEomyV6.35740 | TRI27_HUMAN |
| 17 | 31061881 | 31075378 | 13497 | + | CIGENEomyV6.58929 | LPIN2_MOUSE |
| 21 | 17748164 | 17752489 | 4325 | - | CIGENEomyV6.69302 | F263_HUMAN |
| 21 | 17752725 | 17766922 | 14197 | - | CIGENEomyV6.69303 | SPF45_HUMAN |
| 21 | 17768327 | 17804146 | 35819 | + | CIGENEomyV6.69304 | I15RA_MOUSE |
| 22 | 42897113 | 42898063 | 950 | - | CIGENEomyV6.72362 | TAA8B_MOUSE |
| 24 | 13381639 | 13383797 | 2158 | + | CIGENEomyV6.75078 | INT4_HUMAN |
| 24 | 13382893 | 13393857 | 10964 | - | CIGENEomyV6.75079 | PANX1_MOUSE |
| 24 | 13402168 | 13417551 | 15383 | + | CIGENEomyV6.75081 | DCLK1_HUMAN |
| 25 | 61663326 | 61713003 | 49677 | + | CIGENEomyV6.79021 | 2A5E_HUMAN |
| 25 | 61739034 | 61740259 | 1225 | - | CIGENEomyV6.79022 | GPHB5_MOUSE |
| 28 | 6177592 | 6185474 | 7882 | - | CIGENEomyV6.84115 | NCOA6_MOUSE |
| 28 | 6257262 | 6367954 | 110692 | + | CIGENEomyV6.84111 | S26A6_MOUSE |
| 28 | 6334311 | 6379093 | 44782 | - | CIGENEomyV6.84118 | S26A5_MOUSE |
| 28 | 9037784 | 9045101 | 7317 | - | CIGENEomyV6.84247 | TCB1_CAEBR |
| 28 | 9066715 | 9074417 | 7702 | + | CIGENEomyV6.84250 | CASP8_MOUSE |
| 28 | 9070181 | 9090946 | 20765 | - | CIGENEomyV6.84251 | CASP8_HUMAN |
| 28 | 9103667 | 9109099 | 5432 | - | CIGENEomyV6.84255 | MCM6_HUMAN |
| 28 | 9140216 | 9156842 | 16626 | - | CIGENEomyV6.84260 | TGM1_CANFA |
| 28 | 10449344 | 10452472 | 3128 | + | CIGENEomyV6.84347 | RHG30_HUMAN |
| 28 | 10452613 | 10461808 | 9195 | + | CIGENEomyV6.84349 | ZG67_XENLA |
| 28 | 10456921 | 10468409 | 11488 | - | CIGENEomyV6.84350 | ZN613_HUMAN |
| 28 | 10477161 | 10695364 | 218203 | + | CIGENEomyV6.84351 | JUN_SERCA |
| 28 | 10484458 | 10505199 | 20741 | - | CIGENEomyV6.84352 | GPBL1_RAT |
| 28 | 10499461 | 10505199 | 5738 | + | CIGENEomyV6.84353 | TMM69_XENTR |
| 28 | 10515713 | 10523214 | 7501 | - | CIGENEomyV6.84354 | P55G_BOVIN |
| 28 | 10594106 | 10606086 | 11980 | - | CIGENEomyV6.84356 | MGST3_BOVIN |
| 28 | 10640906 | 10662184 | 21278 | - | CIGENEomyV6.84357 | ANGL1_BOVIN |

| **CHR** | **5' pos** | **3' pos** | **Length (bp)** | **Ori.** | **Gene ID** | **Gene** |
| --- | --- | --- | --- | --- | --- | --- |
| 28 | 10723658 | 10724777 | 1119 | + | CIGENEomyV6.84359 | RTJK_DROME |
| 28 | 10742437 | 10777969 | 35532 | + | CIGENEomyV6.84360 | SUCO_RAT |
| 28 | 10959954 | 10970333 | 10379 | + | CIGENEomyV6.84367 | LPP3_BOVIN |
| 28 | 11014022 | 11034719 | 20697 | + | CIGENEomyV6.84369 | ODFP2_CHICK |
| 28 | 11038306 | 11041755 | 3449 | - | CIGENEomyV6.84370 | CYR61_CHICK |
| 28 | 11068746 | 11070498 | 1752 | + | CIGENEomyV6.84371 | BCL10_RAT |
| 28 | 11075232 | 11081759 | 6527 | + | CIGENEomyV6.84373 | MCLN3_MOUSE |
| 28 | 11084074 | 11129693 | 45619 | + | CIGENEomyV6.84375 | ADIP_ORYLA |
| 28 | 11097926 | 11101144 | 3218 | - | CIGENEomyV6.84376 | SAM13_HUMAN |
| 28 | 11139254 | 11155396 | 16142 | - | CIGENEomyV6.84378 | PTER_SALSA |
| 28 | 11170607 | 11214751 | 44144 | - | CIGENEomyV6.84379 | MMP16_HUMAN |
| 28 | 11228399 | 11288483 | 60084 | + | CIGENEomyV6.84380 | EGFR_MOUSE |
| 28 | 11290774 | 11337500 | 46726 | + | CIGENEomyV6.84382 | NPC1_PIG |
| 28 | 11323708 | 11360085 | 36377 | - | CIGENEomyV6.84383 | CABL1_HUMAN |
| 28 | 11405777 | 11415757 | 9980 | - | CIGENEomyV6.84387 | GAT6A_XENLA |
| 28 | 11482532 | 11569745 | 87213 | - | CIGENEomyV6.84395 | MIB1_DANRE |
| 28 | 11574784 | 11602912 | 28128 | + | CIGENEomyV6.84397 | ABHD3_HUMAN |
| 28 | 11599086 | 11650897 | 51811 | - | CIGENEomyV6.84398 | GRB1L_HUMAN |
| 28 | 11716664 | 11791337 | 74673 | + | CIGENEomyV6.84402 | ROCK1_RABIT |
| 28 | 11791778 | 11807721 | 15943 | + | CIGENEomyV6.84403 | UBP14_RABIT |
| 28 | 11808202 | 11817079 | 8877 | - | CIGENEomyV6.84404 | THOC1_HUMAN |
| 28 | 11832995 | 11864414 | 31419 | - | CIGENEomyV6.84406 | AQPA_PELES |
| 28 | 12014619 | 12264131 | 249512 | + | CIGENEomyV6.84408 | CRFR2_XENLA |
| 28 | 12110380 | 12119079 | 8699 | - | CIGENEomyV6.84409 | MYL3_MOUSE |
| 28 | 12222862 | 12249020 | 26158 | + | CIGENEomyV6.84410 | PTH1R_DIDVI |
| 28 | 12268341 | 12288985 | 20644 | - | CIGENEomyV6.84413 | TMIE_MOUSE |
| 28 | 12300397 | 12311401 | 11004 | - | CIGENEomyV6.84414 | CCD13_HUMAN |
| 28 | 12320175 | 12334008 | 13833 | + | CIGENEomyV6.84415 | CP8B1_HUMAN |
| 28 | 12335161 | 12361202 | 26041 | + | CIGENEomyV6.84417 | CXG1_HUMAN |
| 28 | 12361588 | 12365670 | 4082 | - | CIGENEomyV6.84418 | TCB2_CAEBR |
| 28 | 12374700 | 12379679 | 4979 | - | CIGENEomyV6.84419 | JMJD4_DANRE |
| 28 | 12378325 | 12385147 | 6822 | + | CIGENEomyV6.84420 | SNP47_DANRE |
| 28 | 12565706 | 12599594 | 33888 | - | CIGENEomyV6.84424 | WNT9A_CHICK |
| 28 | 12666331 | 12694559 | 28228 | + | CIGENEomyV6.84427 | ARF1_XENLA |

**Table S2.** Annotated genes that fall within significantly differentiated genomic regions according to local score analyses on Kalama and Klickitat premature vs. mature libraries. The 5’ and 3’ locations of each gene are denoted along with the orientation.

| **Mis.** | **Chr** | **Pos** | **-log(10)p** | **Depth (X)** | **MapQ** | **Nuc.** | **Amino Acid** | **Pos.** | **Association** |
| --- | --- | --- | --- | --- | --- | --- | --- | --- | --- |
| 1 | 28 | 11606585 | 5.04 | 126 | 60 | G | Alanine (Ala) | 1 | Mature |
|  |  |  |  |  |  | A | Threonine (Thr) |  | Premature |
| 2 | 28 | 11607954 | 11.37 | 116 | 60 | T | Cysteine (Cys) | 1 | Mature |
|  |  |  |  |  |  | C | Arginine (Arg) |  | Premature |
| 3 | 28 | 11622315 | 11.78 | 96 | 60 | T | Tyrosine (Tyr) | 1 | Mature |
|  |  |  |  |  |  | G | Aspartic acid (Asp) |  | Premature |
| 4 | 28 | 11632591 | 14.34 | 119 | 59 | C | Leucine (Leu) | 1 | Mature |
|  |  |  |  |  |  | T | Phenylalanine (Phe) |  | Premature |
| 5 | 28 | 11646420 | 12.29 | 104 | 60 | T | Valine (Val) | 2 | Mature |
|  |  |  |  |  |  | A | Aspartic acid (Asp) |  | Premature |

**Table S3.**  Five non-synonymous SNPs (nsSNPs) determined within the differentiated 309-kb region on chromosome 28. All nsSNPs fall within greb1L, but only the latter four are significant ( -log[10]p > 10). Quality statistics, such as the total depth of coverage at the genomic position (Depth [X]), the mapping quality score (MapQ) are provided. The nucleotide substitution (Nuc.) position in the codon (Pos.) are also provided.

| **ID** | **PID** | **Name** | **Sub-Basin** | **Maturation Type** | **Long** | **Lat** | **N** | **Clust** | **(Pre) AA** | **(Het) AG** | **(Mat) GG** |
| --- | --- | --- | --- | --- | --- | --- | --- | --- | --- | --- | --- |
| 1 | ABER | Abernathy | Elochoman | Ocean | -123.15 | 46.23 | 21 | COA | 0.0% | 6.3% | 93.8% |
| 2 | KALS | Kalama - Summer | Kalama | Stream | -122.87 | 46.03 | 46 | COA | 67.5% | 15.0% | 17.5% |
| 3 | KALW | Kalama - Winter | Kalama | Ocean | -122.87 | 46.03 | 36 | COA | 3.3% | 0.0% | 96.7% |
| 4 | NLEW | North Fork Lewis | Lewis | Ocean | -122.56 | 45.96 | 40 | COA | 5.9% | 2.9% | 91.2% |
| 5 | ELEW | East Fork Lewis | Lewis | Unk. Mix | -122.78 | 45.85 | 38 | COA | 37.0% | 14.8% | 48.1% |
| 6 | WHOO | West Fork Hood | Hood | Stream | -121.69 | 45.56 | 45 | COA | 84.1% | 13.6% | 2.3% |
| 7 | EAGL | Eagle | Willamette | Ocean | -122.38 | 45.35 | 46 | COA | 2.5% | 12.5% | 85.0% |
| 8 | HOOH | Hood | Hood | Ocean | -121.62 | 45.52 | 46 | COA | 0.0% | 9.3% | 90.7% |
| 9 | SKAM | Skamania Stock | Willamette * | Stream | -122.28 | 45.24 | 47 | COA | 100.0% | 0.0% | 0.0% |
| 10 | WWIL | West Willamette | Willamette | Ocean | -123.15 | 44.75 | 44 | COA | 6.1% | 3.0% | 90.9% |
| 11 | NSAN | Little Rock | Willamette | Unk. Mix | -122.40 | 44.75 | 28 | COA | 50.0% | 22.2% | 27.8% |
| 12 | SSAN | South Santiam | Willamette | Unk. Mix | -122.67 | 44.42 | 31 | COA | 24.0% | 8.0% | 68.0% |
| 13 | EFHJ | East Fork Hood | Hood | Ocean | -121.59 | 45.56 | 46 | COA | 9.8% | 14.6% | 75.6% |
| 14 | KLIS | Klickitat - Summer | Klickitat | Stream | -121.26 | 45.72 | 132 | COA* | 75.4% | 15.6% | 9.0% |
| 15 | KLIW | Klickitat - Winter | Klickitat | Ocean | -121.26 | 45.72 | 99 | COA* | 15.1% | 8.6% | 76.3% |
| 16 | WNFH | Winthrop | Methow | Stream | -120.19 | 48.48 | 47 | INT | 42.2% | 46.7% | 11.1% |
| 17 | OKAN | Okanogan | Methow | Stream | -119.59 | 48.38 | 47 | INT | 38.6% | 40.9% | 20.5% |
| 18 | LIBB | Methow | Methow | Stream | -120.11 | 48.23 | 18 | INT | 12.5% | 18.8% | 68.8% |
| 19 | CHIW | Chiwaukum | Wenatchee | Stream | -120.74 | 47.69 | 34 | INT | 50.0% | 15.4% | 34.6% |
| 20 | NACH | Naches -Nile | Yakima | Stream | -121.05 | 46.86 | 46 | INT | 20.5% | 13.6% | 65.9% |
| 21 | SATU | Satus | Yakima | Stream | -120.61 | 46.20 | 47 | INT | 2.1% | 14.9% | 83.0% |
| 22 | YAKC | Yakima | Yakima | Stream | -119.76 | 46.22 | 200 | INT | 2.7% | 24.9% | 72.4% |
| 23 | ROCK | Rock | Mid. Columbia | Stream | -120.44 | 45.75 | 40 | INT | 18.9% | 18.9% | 62.2% |
| 24 | MILL | Mill Creek | Mid. Columbia | Stream | -121.19 | 45.61 | 46 | INT | 26.1% | 34.8% | 39.1% |
| 25 | FIFT | Fifteen | Fifteenmile | Stream | -121.13 | 45.51 | 47 | INT | 0.0% | 17.4% | 82.6% |
| 26 | DESC | Deschutes | Deschutes | Stream | -121.28 | 44.61 | 200 | INT | 10.6% | 44.4% | 45.0% |
| 27 | JDMA | John Day Main | John Day | Stream | -119.57 | 44.33 | 80 | INT | 1.4% | 2.8% | 95.8% |
| 28 | SFJD | South John Day | John Day | Stream | -119.12 | 44.41 | 30 | INT | 0.0% | 0.0% | 100.0% |
| 29 | MFJD | Middle John Day | John Day | Stream | -118.51 | 44.59 | 47 | INT | 2.4% | 4.9% | 92.7% |

| **ID** | **PID** | **Name** | **Sub-Basin** | **Maturation Type** | **Long** | **Lat** | **N** | **Clust** | **(Pre) AA** | **(Het) AG** | **(Mat) GG** |
| --- | --- | --- | --- | --- | --- | --- | --- | --- | --- | --- | --- |
| 30 | NFJD | Upper John Day | John Day | Stream | -118.48 | 44.84 | 46 | INT | 0.0% | 7.7% | 92.3% |
| 31 | MINT | Minthorn | Umatilla | Stream | -118.62 | 45.67 | 47 | INT | 4.5% | 13.6% | 81.8% |
| 32 | UMAT | Umatilla | Umatilla | Stream | -118.40 | 45.70 | 46 | INT | 4.8% | 11.9% | 83.3% |
| 33 | TUCN | Tucannon | Tucannon | Stream | -117.66 | 46.31 | 46 | INT | 8.1% | 16.2% | 75.7% |
| 34 | ASOT | Asotin | Asotin | Stream | -117.14 | 46.32 | 45 | INT | 34.9% | 25.6% | 39.5% |
| 35 | MISS | Mission | Clearwater | Stream | -116.74 | 46.37 | 40 | INT | 13.2% | 23.7% | 63.2% |
| 36 | EPOT | East Potlatch | Clearwater | Stream | -116.42 | 46.80 | 34 | INT | 0.0% | 19.4% | 80.6% |
| 37 | DWOR | Dworshak | Clearwater | Stream | -116.33 | 46.50 | 27 | INT | 0.0% | 7.7% | 92.3% |
| 38 | CAPJ | Captain John | Snake | Stream | -116.93 | 46.15 | 43 | INT | 13.2% | 44.7% | 42.1% |
| 39 | JOSE | Joseph | Grande Ronde | Stream | -117.02 | 46.03 | 46 | INT | 13.6% | 34.1% | 52.3% |
| 40 | UGRT | Grande Ronde | Grande Ronde | Stream | -117.86 | 45.73 | 43 | INT | 2.5% | 20.0% | 77.5% |
| 41 | CATH | Catherine | Grande Ronde | Stream | -117.87 | 45.31 | 36 | INT | 8.8% | 8.8% | 82.4% |
| 42 | LSHE | LittleSheep | Imnaha | Stream | -116.93 | 45.48 | 37 | INT | 8.6% | 5.7% | 85.7% |
| 43 | IMNA | Cow | Imnaha | Stream | -116.75 | 45.77 | 14 | INT | 8.3% | 16.7% | 75.0% |
| 44 | WHIT | Whitebird | Salmon | Stream | -116.32 | 45.75 | 44 | INT | 23.8% | 42.9% | 33.3% |
| 45 | TENM | Tenmile | Clearwater | Stream | -115.68 | 45.81 | 41 | INT | 0.0% | 0.0% | 100.0% |
| 46 | CROO | Crooked | Clearwater | Stream | -115.53 | 45.82 | 45 | INT | 2.5% | 2.5% | 95.0% |
| 47 | FISH | Fish/Lochsa | Clearwater | Stream | -115.35 | 46.33 | 47 | INT | 0.0% | 0.0% | 100.0% |
| 48 | LAKE | Lake/Lochsa | Clearwater | Stream | -115.00 | 46.46 | 42 | INT | 0.0% | 5.4% | 94.6% |
| 49 | BOUL | Boulder | Clearwater | Stream | -114.74 | 46.68 | 47 | INT | 0.0% | 0.0% | 100.0% |
| 50 | EMOO | East Moose | Clearwater | Stream | -114.90 | 46.19 | 40 | INT | 0.0% | 7.9% | 92.1% |
| 51 | LCLW | Little Clearwater | Clearwater | Stream | -114.77 | 45.75 | 47 | INT | 2.2% | 4.3% | 93.5% |
| 52 | BARG | Bargamin | Salmon | Stream | -115.19 | 45.57 | 24 | INT | 60.0% | 20.0% | 20.0% |
| 53 | CHAM | Chamberlain | Salmon | Stream | -114.93 | 45.45 | 48 | INT | 65.9% | 31.8% | 2.3% |
| 54 | LICK | Lick Creek | Salmon | Stream | -115.76 | 45.06 | 43 | INT | 44.1% | 0.0% | 55.9% |
| 55 | LOON | Loon | Salmon | Stream | -114.81 | 44.81 | 45 | INT | 83.7% | 14.0% | 2.3% |
| 56 | PAHH | Salmon | Pahsimeroi | Stream | -114.03 | 44.66 | 46 | INT | 15.4% | 11.5% | 73.1% |
| 57 | MARS | Marsh | Salmon | Stream | -115.23 | 44.45 | 45 | INT | 73.2% | 19.5% | 7.3% |
| 58 | WFYF | Yankee Fork | Salmon | Stream | -114.73 | 44.35 | 46 | INT | 45.0% | 47.5% | 7.5% |
| 59 | SAWN | Sawtooth | Salmon | Stream | -114.88 | 44.15 | 41 | INT | 5.1% | 25.6% | 69.2% |

**Table S4.** List of 59 collection localities genotyped at the diagnostic greb1L SNP. The known phenotypic maturation at fresh water entry (either stream-maturing or ocean-maturing) is provided along with if the population belongs to the interior (INT) or coastal (COA) genetic lineage. The proportion of maturity genotypes denote the proportion of genetically mature (GG), premature (AA), and heterozygote (AG) fish in each population.

|  |  |  |  |  | Difference of arrival week mean | | | | Tukey's p-value | | |
| --- | --- | --- | --- | --- | --- | --- | --- | --- | --- | --- | --- |
| Sub-basin | n Pre. | n Het. | n Mat. | **Var. Exp.** | **Pre. vs. Het.** | **Mat. vs. Het.** | **Pre. Vs. Mat** | **Pr(>F)** | **Pre. vs. Het.** | **Mat. vs. Het.** | **Pre. Vs. Mat** |
| Klickitat | 18 | 21 | 20 | 16.77% | 0.56 | 2.16 | 2.73 | **0.005** | 0.78 | **0.032** | **0.007** |
| Yakima | 9 | 47 | 92 | 10.58% | 3.69 | 1.03 | 4.72 | **< 0.001** | **0.008** | 0.21 | **< 0.001** |
| Tucannon | 9 | 25 | 57 | 6.30% | 0.35 | 1.78 | 1.43 | 0.057 | 0.94 | 0.06 | 0.43 |
| Asotin | 34 | 58 | 79 | 12.37% | 1.53 | 0.5 | 2.04 | **0.012** | 0.084 | 0.66 | **0.008** |
| Grande Ronde | 11 | 54 | 156 | 4.05% | 1.37 | 1.17 | 2.54 | **0.011** | 0.44 | 0.078 | **0.047** |
| Clearwater | 8 | 38 | 154 | 12.37% | 2.34 | 2.44 | 4.78 | **< 0.001** | 0.184 | **< 0.001** | **< 0.001** |
| **Mean** | 14.83 | 40.5 | 93 | 10% | 1.64 | 1.51 | 3.04 |  |  |  |  |

**Table S5.** Genotypes and their association with spawning week in six sub-basins containing spawning headwater tributaries with PIT-tag arrays. The number of fish with each maturation genotype (premature [Pre.=AA], mature [Mat.=GG], and heterozygote [Het.=AG]) are noted along with the variance of spawning time week explained by genotype, based on the sum of squares of a one-way ANOVA. The mean difference in spawning week between each genotype class and the significance of the means being unequal (F-value) are provided. Finally, pairwise significance of mean spawning week between each genotype class (premature, heterozygote, and mature) from a Tukey’s test.

**Additional File 1: Sequencing Protocol**

**Pool-Seq – Modified NEBNEXT Protocol**

1. *Optional*: Run 2% Agarose gel on DNA – check DNA quality
2. **Quantify DNA** using M200 1.20 dilution *(See Appendix A)*
   1. May need to use 1.100 dilution for extractions yielding more DNA (E.g., Chelex)
3. **Normalize DNA**: Target 500ng-1000ng genomic DNA in 200ul total volume
4. **Quantify Normalized DNA** using M200 (*Post Norm Quantification* -*See Appendix A)*
   1. Ensure no individuals are under or overrepresented
   2. Set a threshold of +/-20% around the mean *(See Appendix B)*
      1. For example, if the post-norm mean for a library is 4 ng/uL, ; 4 x .2 = 0.8; therefore we require that all individuals fall between 3.2 and 4.8 ng/uL. This also means the maximum variation between samples allowed is 66% (3.2/4.8).
   3. Some samples may have to be adjusted by the above guidelines, therefore you must perform a post post normalization to ensure the adjustment was successful.
5. **Pool Normalized DNA**
   1. Again, we are targeting 500ng-1000ng genomic DNA in 200ul total volume.
      1. For example:
      2. 40 individuals in 1 library
      3. Normalize each individual to 5ng
      4. Pool 5ul DNA (5ng)*40 individuals = 200ul pooled volume
      5. 5ng DNA per individual *5ul DNA = 25ng DNA per individual…..25ng DNA * 40 individuals = 1000ng DNA total in 200ul pooled volume
6. **Fragment pooled DNA**
   1. Do **4** 50ul digests using NEBNext dsDNA Fragmentase in 4 separate wells of low profile plate.
      1. 40ul Pooled DNA
      2. 5ul 10x Fragmentase Reaction Buffer v2
      3. 5ul dsDNA Fragmentase
      4. 50ul total volume x 4 wells = 200ul total volume
   2. Incubate at 37ºC for 10 minutes on thermalcycler & ***remove promptly***.
   3. ***Immediately*** add 12.5ul 0.5M EDTA to all 4 wells to stop the reaction.
7. **Pool fragmented DNA** into 1 tube
   1. Total volume = 250ul
8. **Clean pooled fragmented DNA** using Minelute Purification.
   1. Heat Buffer EB to 55°C
   2. Add 5 times the volume of **Buffer PB** to pooled/fragmented DNA
      1. 5 x 250ul = 1250ul Buffer PB
      2. Mix well, but **DO NOT CENTRIFUGE**
   3. Put 750ul or less at a time of library into 1 Minelute column.
      1. Use vacuum to filter through column. Continue until total library volume is filtered.
   4. To wash, add 750ul **Buffer PE** to Minelute column.
      1. Use vacuum to filter through column.
   5. Centrifuge Minelute column @ 1000 rcf for 10 seconds with the tube lid open.
   6. Close the Minelute column lid and centrifuge @16,100 rcf for 1 minute.
   7. Place column in a clean 1.5ml microcentrifuge tube
   8. To elute DNA, pipette 62ul of **Buffer EB** to the center of the membrane and let column stand for 1 minute
   9. Centrifuge @10,000 rcf for 1 minute
9. **NEBNext End Prep**
   1. Mix the following in a pcr plate:
      1. 3.0ul End Prep Enzyme Mix
      2. 6.5ul End Repair Reaction Buffer (10X)
      3. 55.5ul Fragmented & Purified DNA
   2. Place in thermalcycler:
      1. 30 minutes @ 20ºC
      2. 30 minutes @ 65ºC
      3. Hold at 4ºC
10. **Adaptor Ligation**
    1. Add the following directly to the End Prep reaction mixture and mix well:
       1. 15ul Blunt/TA Ligase Master Mix
       2. 2.5ul NEBNext Adaptor for Illumina
       3. 1ul Ligation Enhancer
    2. Incubate in thermalcycler at 20ºC for 15 minutes.
    3. Add 3ul USER enzyme to the ligation mixture
    4. Mix well and incubate on thermalcycler at 37ºC for 15 minutes.
11. **Size Selection of Adaptor Ligated DNA (Target 400-500bp (Approx. Insert Size))**
    1. Vortex AMPure Beads
    2. Add 13.5ul DH2O to the ligation reaction for a 100ul total volume.
    3. Transfer total volume to strip tube
    4. Add 35ul of resuspended AMPure XP Beads, mix well and incubate at room temperature for 5 minutes.
    5. Place on the magnetic stand for 5 minutes.
    6. Transfer supernatant to a new tube and remove from magnetic stand.
    7. Add 15ul of resuspended AMPure XP Beads, mix well and incubate at room temperature for 5 minutes.
    8. Place on the magnetic stand for 5 minutes.
    9. Remove and discard supernatant.
    10. Add 200ul of 80% freshly prepared ethanol to the tube while in the magnetic stand. Incubate at room temperature for 30 seconds, and then carefully remove and discard the supernatant.
    11. Repeat previous step once.
    12. Air dry the beads for 5 minutes while the tube is on the magnetic stand. **CAUTION: Do not overdry the beads – this may result in lower recovery of DNA target.**
    13. Remove tube from magnetic stand & elute with 17ul 1xTE. Mix well and incubate at room temperature for 2 minutes.
    14. Place on magnetic stand for 5 minutes and collect supernatant.
12. **PCR Amplification**
    1. Mix the following in a pcr tube:
       1. 15ul Adapter Ligated DNA Fragments
       2. 25ul NEBNext Q5 Hot Start HiFi PCR Master Mix
       3. 5ul i7 Index (10uM)
       4. 5ul Universal PCR Primer
    2. Place on thermalcycler:
       1. 98.0°-00:30, (98.0°-00:10, 65.0°-00:75)-5 cycles and (65.0°-5:00, 4.0°-hold).
13. **Cleanup of PCR Amplification**
    1. Transfer total volume to strip tube
    2. Vortex AMPure Beads
    3. Add 45ul resuspended AMPure XP Beads to the PCR reaction, mix well and incubate at room temperature for 5 minutes.
    4. Place on magnetic stand for 5 minutes.
    5. Remove and discard supernatant.
    6. Add 200ul of 80% freshly prepared ethanol to the tube while in the magnetic stand. Incubate at room temperature for 30 seconds, and then carefully remove and discard the supernatant.
    7. Repeat previous step once.
    8. Air dry the beads for 5 minutes while the tube is on the magnetic stand. **CAUTION: Do not overdry the beads – this may result in lower recovery of DNA target.**
    9. Remove tube from magnetic stand & elute with 33ul 1xTE. Mix well and incubate at room temperature for 2 minutes.
    10. Place on magnetic stand for 5 minutes and collect supernatant.
14. **qPCR**
    1. Dilute samples 1:1000 by taking 1998µL of TE/TWEEN and putting it into a 2.0mL micro tube. Put 2µL of library sample into micro tube. To accurately dispense, use non-filter tips; prime tip several times by drawing up and dispensing sample. Rinse tip after dispensing in micro tube by drawing up and dispensing several times. Vortex and spin down.
    2. In low profile PCR plate make further dilutions of 1:2000, 1:4000, and 1:8000 by taking 100µL of the 1:1000 sample dilution and putting it into the first row wells of the plate using a separate column for each library. In the 2nd, 3rd, and 4th, rows put 50µL of TE/TWEEN. Take 50µL from the first row and dispense into the second row. Mix well by pipetting up and down 6-7 times. Make sure tips are completely voided and take 50µL of the second row and transfer into the third row, mixing as before. Finally take 50µL of the third row and transfer to the fourth row, and mix well. Take 50µL from the fourth row and discard.
    3. In an ABI clear 384 well plate set up the plate by using 3 columns for the 6 standards. Use RAD standards.
    4. In the same 384 well plate, use 1 column for each library with 2 rows for each of the dilutions, for a total of 8 rows. Use a 30µL matrix pipette to draw up 10.3µL of the sample column from step 2 and dispense 5µL into the first and second rows. Discard the remaining 0.3µL in the pipette tips.
    5. Make primer mix with FAST SYBR green. Take the number of libraries and times them by the number of rows. Add the number of standard samples that you have to get total samples. (7 libraries X 8 rows of dilutions=56. 3 columns of 6 standards=18. 56+18=74 over estimate to 80 total samples).

**Reagent Mix X1 X80**

SYBR Fast Green Master Mix (2X) 7.5µL 600µL

Illumina Primer Mix (10X) 1.5µL 120µL

TE/TWEEN 1.0µL 80µL

Total 10µL 800µL

DNA volume per well 5.0 µL -------

- 1. Add 10µL of Reagent Mix to all the standard and library wells using a repeating pipette. Dispense gently to ensure no splashing. Put kimwipe under plate and spin down in centrifuge. Check for bubbles in wells. If bubbles are present, pop with the bubble popper 3000.
  2. Put an ABI Micro Amp Optical Adhesive Film on the plate. Use clean edge of PCR plate to ensure good contact. Pull off tabs of cover.
  3. Run qPCR on Quant Studio 6 Flex
  4. Minimum concentration to proceed is 2.5nM

1. **Run final library on Bioanalyzer** using DNA High Sensitivity Chip *(See Appendix C)*
   1. Target 400-500bp.
   2. Bioanalyzer user manual is kept in drawer below instrument.
2. **Normalize final library** to 4nM and add to sequencing queue.
   1. **When pooling two Pool Seq libraries together, use equal volumes. Also, be sure the i7 indexes are compatible.

Appendix A

Step 1. Quantify DNA

Sample Dilutions (1:20)

1. Defrost frozen samples at 37C as needed, and pipette mix
2. Pre-dilute samples if there is less than 20ul original sample (i.e. bring up volume to ~50 ul)
3. Add 76ul of TE/TWEEN buffer (50ml TE, 50**ul** Tween-20) to each well (38ul for limited DNA)
4. Add 4ul of each sa**m**ple, mix 2x after dispensing to transfer whole volume (2ul for limited DNA)
5. Heat seal, vortex and spin down

Standards Dilutions

1. In tube 1 of 8-strip tube, add 72ul of TE/TWEEN. (100,000/10 = 10,000 ng/ml )
2. In tubes 2-8 add 40ul of TE/TWEEN
3. In tube 1 add 8ul Standard, mix 2x after dispensing to transfer whole volume
4. Set pipette at 40 ul, mix tube 1 using 6 aspiration/dispense cycles
5. Take 40ul from first row, and add to second row, mixing 6 times
6. Repeat with each additional row. (last row will be left with 80ul total volume)

Pico Reagents Mix

Mix Pico green with TE/TWEEN buffer dependent upon sample number. Multiplier assumes ~10% extra plus 32 (standards)

| n | 1 |  | 32 | 48 | 96 | 192 | 288 | 384 |
| --- | --- | --- | --- | --- | --- | --- | --- | --- |
| multiplier |  |  | 72 | 88 | 140 | 244 | 352 | 456 |
| TE/TWEEN ul | 89.5 |  | 6,444 | 7,876 | 12,530 | 21,838 | 31,504 | 40,812 |
| Pico Green ul | 0.5 |  | 36 | 44 | 70 | 122 | 176 | 228 |

Quantification Plates

1. Add 90ul of Pico Reagent mix to each well in black Corning 96-well half area optical plate
2. Add 10ul of diluted standards or samples, and 10ul of TE/TWEEN to blanks (NTC controls) to appropriate wells
3. Cover and incubate at room temperature for 10 minutes.
4. Remove bubbles from plate prior to scan

Tecan M200 analysis

1. Turn on power to computer/Turn on Instrument/Start icontrol software/ *Select Initialize on GUI if needed*
2. *Select Standard tab in lower left hand corner-Should be the default*
3. Open “picogreen manual gain” under File-recent leaks *(File/Open/Shared Docs/Tecan/icontrol/1.1/scripts)*
4. *Check settings ExW=485, EmW=535, ExB=9, EmB=20, Gai=85,Fla=25, InT=20, LaT=0, SeT=0, ZPo=21000, Lab=Picogreen*
   - 1. *Alt Protocol. Use Optimal gain/Zpo on standards plate, then apply as manual for the samples*
5. Add plate to tray.
6. Select the green button for run, wait ~ 1minute

Appendix A (cont.)

1. Save excel file as needed. Multiple scans (e.g. 1 standards plate, 1 samples plate) will create multiple worksheets/tabs. Rename each worksheet as needed to differentiate scans. Do not rename during scan.

Normalization – Best Practices

- Conc. Ng/ml values on the Tecan M200 must be between 8 and 1000 to be considered accurate.
- Poor samples may need a tighter tolerance (20-1000).
- All samples in a single library must be quantified at the same time, using the same standards.
- If performing optional step of **quantifying normalized DNA**: Use 4 ul of normalized DNA, 6 ul TE/Tween, and 90 ul picogreen mix.
- To proceed with a library, 95% of DNA concentrations should be within a two-fold range of each other. E.g., if most of the samples are above 20, most of them should also be below 40.
